# The Expression Levels of Plasma micoRNAs in Atrial Fibrillation Patients

**DOI:** 10.1371/journal.pone.0044906

**Published:** 2012-09-18

**Authors:** Zheng Liu, Cheng Zhou, Yuzhou Liu, Sihua Wang, Ping Ye, Xiaoping Miao, Jiahong Xia

**Affiliations:** 1 Department of Cardiovascular Surgery, Union Hospital, Tongji Medical College, Huazhong University of Science and Technology, Wuhan, China; 2 Department of Cardiovascular Disease, Union Hospital, Tongji Medical College, Huazhong University of Science and Technology, Wuhan, China; 3 Department of Epidemiology and Biostatistics and MOE Key Lab of Environment and Health, School of Public Health, Tongji Medical College, Huazhong University of Science and Technology, Wuhan, China; I2MC INSERM UMR U1048, France

## Abstract

**Background:**

MicroRNA (miRNA) has been found in human blood. It has been increasingly suggested that miRNAs may serve as biomarkers for diseases. We examined the potential of circulating miRNA to serve as predictors of atrial fibrillation (AF).

**Methodology/Principal Findings:**

During the discovery stage of this project, we used massively parallel signature sequencing (MPSS) to carry out an in-depth analysis of the miRNA expression profile (miRNome) in 5 healthy controls, 5 patients with paroxysmal atrial fibrillation (PAF) alone, and 5 patients with persistent atrial fibrillation (PersAF) alone. Twenty-two specific miRNAs were found to be dysregulated in each PAF group, PersAF group, or control group. Four candidate microRNAs (miRNA-146a, miRNA-150, miRNA-19a, and miRNA-375) met our selection criteria and were evaluated in an independent cohort of 90 plasma samples using TaqMan miRNA quantitative reverse transcriptase–polymerase chain reaction (qRT-PCR). We found miRNA-150 levels to be reduced by a factor of approximately 17 in PAF relative to controls and a factor of approximately 20 in PersAF relative to controls (*P*<.0001). Logistic regression analyses were carried out to evaluate the reduced miRNA-150 expression levels (odds ratio [OR] 1.96, 95% confidence interval [CI] 1.5 to 3.57, *P*<0.001), age (OR 1.1, 95% CI 1.36 to 2.73, *P*<0.001), and Left atrial diameter (LAD) (OR 1.5, 95% CI 1.36 to 1.8, *P*<0.001). Each was independently associated with AF. Much of the identified target genes related to AF were part of the inflammatory response system. We found that plasma levels of CRP were negatively correlated with the plasma levels of miRNA-150.

**Conclusions/Significance:**

In summary, we firstly found that plasma miRNA-150 levels in from AF patients were substantially lower than that from healthy people. Circulating reduced miRNA-150 was significantly associated with AF.

## Introduction

Atrial fibrillation (AF) is of great importance to public health. AF causes considerable morbidity, mortality, and health related expenditures. It is anticipated that over the next 4 decades, the prevalence of AF will dramatically increase due to an aging population and improved survival [1], [2]. Despite the fact that the pathophysiology of AF has been investigated extensively for almost a century, the underlying mechanisms remain partially understood. Conventional theories focus on the electrical substrate [3], [4], [5]. In the past few years much attention has been devoted to assess the role of inflammation in AF [6]. Multiple reports suggested that inflammation not only was a marker of incident AF but also was mechanistically involved in the induction of atrial fibrillation [7]. Although there has been steady growth in the number of interventions performed for AF [8], preclinical states that occur as part of the natural history of persistent AF, have not been identified. [9], [10]. Earlier diagnosis of AF with more advantageous monitoring methods could have a significant impact on patient outcomes.

MicroRNAs (miRNAs) are a class of 19–25-nucleotide noncoding RNAs that have been implicated in regulating diverse cellular processes, such as proliferation, differentiation, development, and cell death [11], [12], [13]. In the cardiovascular system, miRNAs play an essential role in hypertrophy, arrhythmia, and ischemia [14], [15], [16], [17], [18]. MiRNAs are thought to play a critical role in regulating the expression of a variety of genes that contribute to AF [16], [19]. However, their potential role as biomarkers for the diagnosis of AF has not yet been systematically evaluated.

Many biomarkers have been assessed for their association with AF. These include N-terminal pro-B-type natriuretic peptide [20], Interleukin-6 [21], [22], osteoprotegerin [23], troponin [24], [25], endothelin [24], and plasminogen activator inhibitor-1 [26]. The relative stability of miRNAs in plasma and serum and the critical roles that miRNAs play in AF suggest broad opportunities for the development of circulating miRNAs as blood-based markers for molecular diagnostics [14], [15], [27], [28].

In the present study we aimed to identify plasma microRNA expression profiles in a cohort of 105 participants, with the intention of identifying microRNAs that were significantly associated with AF. The cohort included healthy individuals and patients with PAF and PersAF.

## Methods

### Study population

Between March 2011 and December 2011, we enrolled 105 participators (61 male, 44 female) with a median age of 51 years. Patients were classified as PAF or PersAF [29]. Every AF patient was monitored using 7-day surface electrocardiography (ECG). Exclusion criteria were age >75 years, hyperthyroidism, uncontrolled hypertension, left ventricular dysfunction with an ejection fraction <40%, malignancy, and acute or chronic inflammatory disease. No patients had structural heart disease. Patients who were undergoing treatment with β-blockers, angiotensin-converting enzyme inhibitors, angiotensin receptor blockers, or statins were enrolled in this study. Among them, 10 patients with a solitary episode of AF (5 with PAF and 5 with PersAF) and 5 controls were selected for the discovery stage for In-depth sequencing of plasma miRNAs levels. The remaining 90 participants were randomly classified as testing using qRT–PCR.

In this study, blood samples were obtained from all enrolled patients. The investigational protocol was approved by the ethics committee of Tongji Medical College, Huazhong University of Science and Technology. Informed consent was obtained from all study participants.

### Definition of Paroxysmal and Persistent AF

PAF was defined as the occurrence in the previous 6 months of 1 or more episodes lasting less than 7 days, with all episodes terminating spontaneously.

PersAF was defined as the occurrence in the previous 12 months of 2 or more episodes of AF, each lasting more than 7 days before being terminated pharmacologically or by electrical cardioversion, or lasting less than 7 days, but necessitating early cardioversion owing to intolerable symptoms or hemodynamic compromise, with sinus rhythm (SR) maintained for 60 minutes or more.

### Plasma collection and storage

Blood samples for miRNA analysis were collected from the patients in the Union Hospital and were processed within 20 min of collection using two-step centrifugation. The supernatant was transferred to RNase/DNase-free tubes and stored at −80°C.

### RNA preparation

Total RNA in plasma was isolated by using a MirVana PARIS kit (Invitrogen) following a modified version of the manufacturer's instructions. *Caenorhabditiselegans* miRNA (cel-miR-39) was synthesized for the spiked-in control [30].

### In-depth sequencing and qRT-PCR

We used massively parallel signature sequencing (MPSS) to carry out an in-depth analysis of the miRNomes in 5 healthy controls, 5 patients with lone PAF, and 5 patients with lone PersAF. cDNA libraries for Solexa/Illumina sequencing were prepared. Briefly, small RNAs were isolated by preparative gel electrophoresis and sequentially ligated to 3′ and then 5′ linkers. Primers complementary to the linker sequences were used for reverse transcription and PCR in order to generate cDNA libraries for deep sequencing. Raw sequencing data were filtered to remove reads lacking identifiable 3′ linker sequences and/or reads falling outside of the predicted miRNA size range. A list of final usable reads was then collapsed down into a list of unique sequences which was analyzed against the samples genome and the mature miRNA database from miRBase (release 9.2) by MegaBLAST using the formatdb, megablast, blastoutparse, and filter alignment scripts of the miRDeep software package. Candidate microRNAs were quantified using the TaqManmiRNA quantitative reverse transcriptase–polymerase chain reaction (qRT–PCR) assay according to the manufacturer's protocol (Applied BioSystems). The assays were performed on 90 samples for 4 candidate miRNAs, miR-19a, miR-146a, hsa-miR-150, and miR-375. The data were analyzed with the automatic setting for assigning baseline. The threshold cycle (Ct) was defined as the fractional cycle number at which fluorescence exceeded the given threshold. The Ct values from real-time PCR assays greater than 40 were treated as 40.

### MiRNA Target Prediction

We predicted miRNA targets using target-prediction programs miRanda, TargetScan, Starbase (Clip-seq) and miRDB. We identified 76 genes from three of four databases. We then used the Database for Annotation, Visualization, and Integrated Discovery (http://david.abcc.ncifcrf.gov) to identify the pathway distribution of predicted targets. These pathways are presented according to the Kyoto Encyclopedia of Genes and Genomes (KEGG) database (http://www.genome.jp/kegg/), which is a database of biological systems, consisting of the genetic building blocks of genes and proteins. The identified pathways involved metabolism, various cellular processes, and human diseases.

### Measurement of hs-CRP

Blood was drawn from an antecubital vein with minimal trauma. The samples were processed using a standardized protocol and stored at 80°C until assayed. The plasma levels of hs-CRP were determined using the Ultrasensitive CRP kit (Abnova. No. KA0238). Results were read at an optical density of 450 nm. Measurements were performed in duplicate, and *P*-values were computed using the two-sided Student t-test (*P*<0.05).

### Statistical analysis

The quantitative data were evaluated for a normal distribution using the Shapiro–Wilk test. The basis for declaring a certain parameter as normally distributed was *P* = 0.20. Normally distributed continuous variables were presented as mean ± SD. Continuous variables that were not normally distributed were presented as medians. Baseline characteristics were assessed using t-tests and Spearman's rank correlation coefficient for continuous variables and χ2 tests for categorical variables. MiRNAs were log-transformed for the multiple logistic regression model in order to improve linear fitting. Logistic regression analyses were performed to identify variables independently associated with expression levels of miRNAs. Results were considered to be statistically significant at *P*<0.05. (SAS version 9.2)

### Statement

The investigational protocol was approved by the ethics committee of Tongji Medical College, Huazhong University of Science and Technology (IRB No: FWA00007304). Informed consent with respective signature was obtained from all study participants, and everyone we recorded fully understood and supported our study. Consent was written by every participant. The following documents were reviewed and approved: 1.protocol; 2.Informed Consent Document; 3. Questionair for Patients.

## Results

### Detection of plasma miRNAs

**Figure 1:**
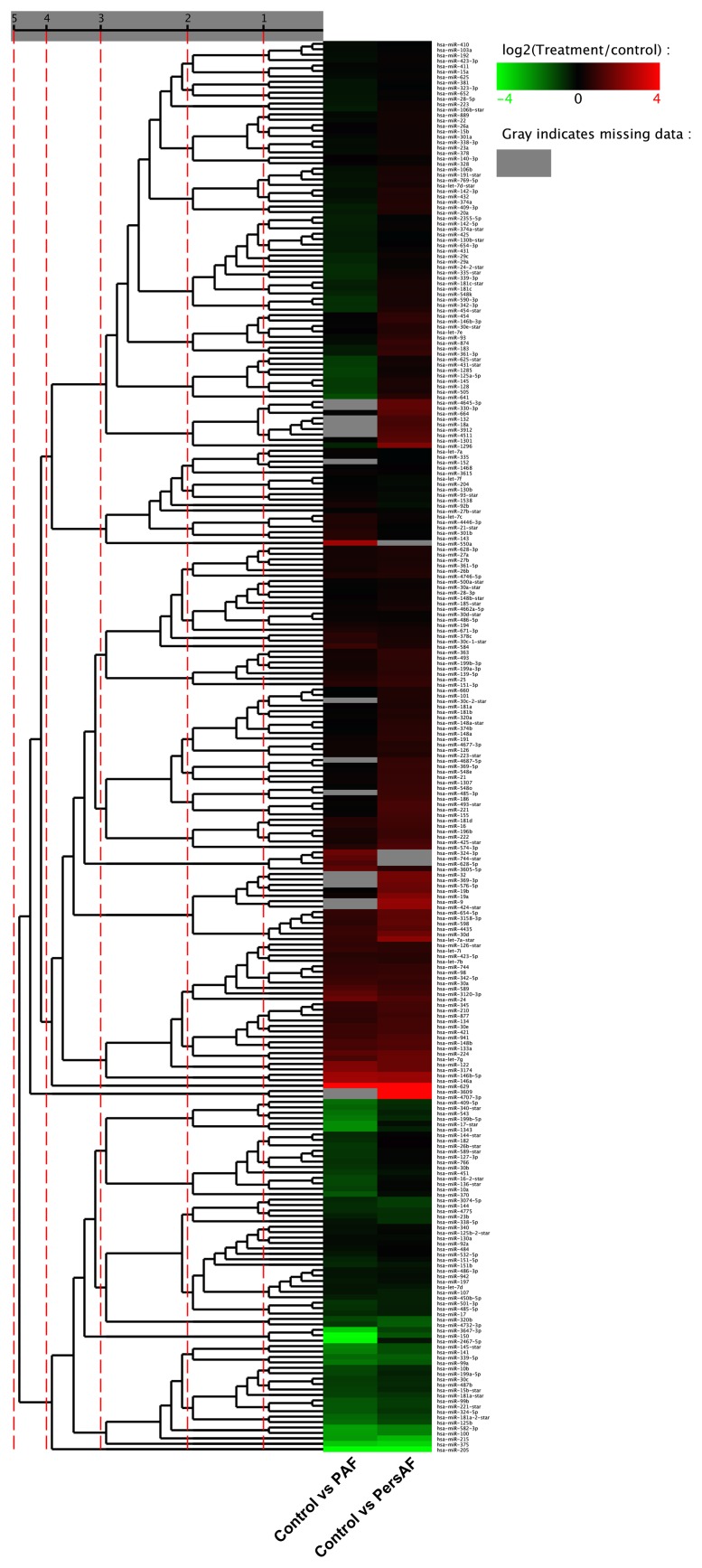
Clusters of circulating miRNAs in patients with PAF or PersAF versus controls. The expression levels of 256 miRNAs by in-depth analysis.

**Figure 2:**
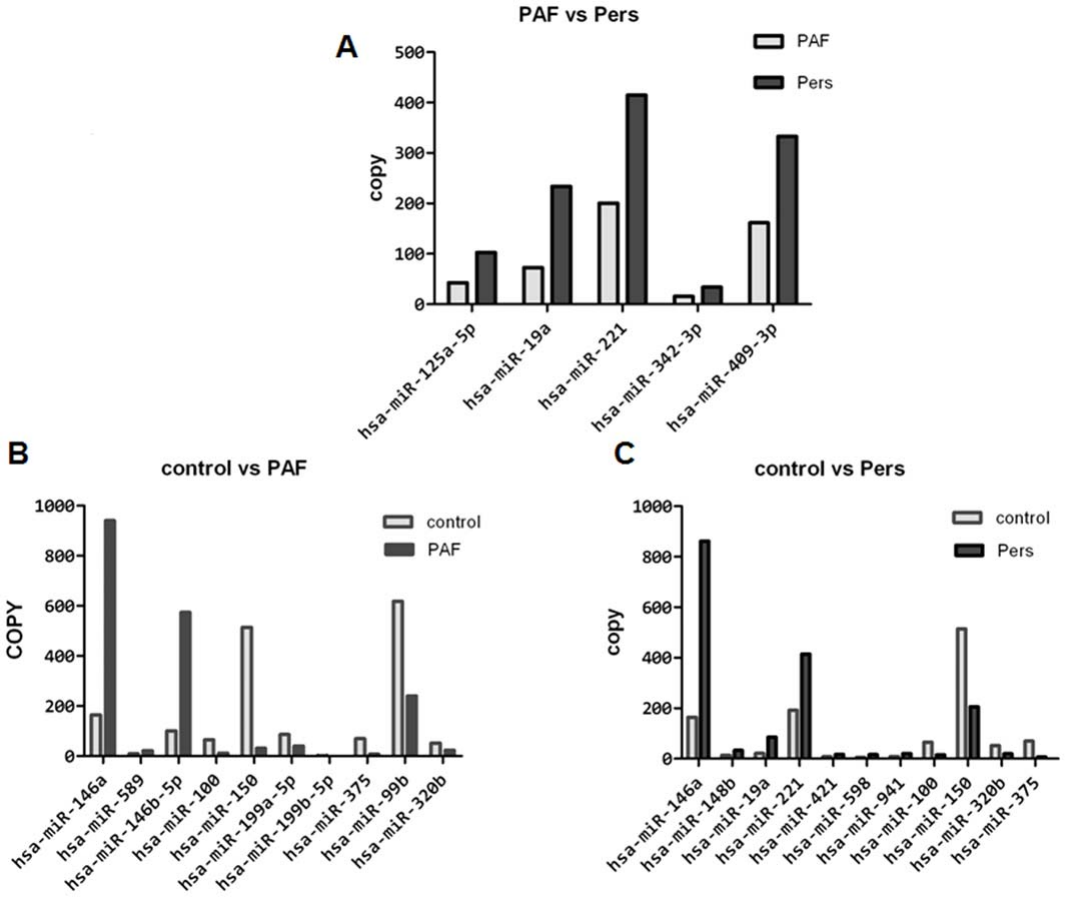
In-depth analysis showed specific miRNAs to be dysregulated in PAF, PeresAF and the control group. Bargraphs showing mean copy values of the miRNAs that satisfied two criteria: having at least 10 copies in either PAF or PersAF groups and showing three-fold altered expression between either of the two pooled samples. (A) Five selected miRNAs in the PAF and PersAF groups. (B) Eleven selected miRNAs in the PAF and the control group. (C) Eleven selected miRNAs in the PersAF and the control group.

In the discovery stage, 243 miRNAs were detected in the PAF group, and 256 miRNAs were detected in the PersAF group (Fig. 1). Five specific miRNAs were found to be upregulated in patients with PAF but not in patients with PersAF. Eleven specific miRNAs were found to be dysregulated between PAF and controls, and another eleven between PersAF and controls (Fig. 2). We selected miRNAs that satisfied the two criteria for additional qRT-PCR validation: at least 10 copies in either PAF or PersAF groups, and showing three-fold altered expression between either two sets of pooled samples [31]. Four plasma miRNAs were identified and subjected to additional analyses. Blood levels of miR-328, which is known to be upregulated in cardiomyocytes after atrial fibrillation [16], were not significantly dysregulated relative to the control group.

We detected the differentially expressed microRNAs in the pairwise comparison of the PAF and healthy groups, PersAF and healthy groups, and AF and PersAF groups, respectively. MiR-146a showed a significantly higher expression level in the AF group, compared to the healthy group (fold change = 5.7 *P*<0.01). In contrast, miR-150 and miRNA-375 had a significantly lower levels of expression in the PAF group than in the healthy group (fold change = 0.06; 0.11 respectively, *P*<0.01). Patients with PersAF had significantly higher levels of miR-146a and miR-19a expression than the control group (fold change = 5.2; 4.0 *P*<0.01). Patients with PersAF had significantly higher levels of miR-150 and miR-19a expression compared to patients with PAF (fold change = 6.6; 3.5 *P*<0.01).

### Patient Characteristics

Thirty control subjects, 30 patients with PAF and 30 patients with PersAF were identified and included in the study (Table 1). The mean age of the PAF patients was 42.5±13.5 years. The mean age of the PersAF was 62±13 years. The mean age of the controls was 37±13 years. The PAF group consisted of 18 men and 12 women. The PersAF group contained 15 men and 15 women. The control group consisted of 20 men and 10 women. Left atrial diameter (LAD) was increased among patients with PAF (50±9 mm) and patients with PersAF (55±8 mm), compared to the control group (43±5 mm, both *P*<0.001).

**Table 1:** Patient Characteristics.

	control group (n = 30)	Paroxysmal AF group (n = 30)	P value	persistent AF group (n = 30)	P value
Age, (y)	37±13	42.6±13.5	0.144	62±13	<.0001
Male sex, n (%)	20(66.67)	18(60.00)	0.6863	15(50.00)	0.4857
Left atrial diameter,(mm)	43±5	50±9	0.001	55±8	<.0001
Hypertension, n (%)	10(33.33)	13(43.33)	0.4857	22(73.33)	0.0137
LVEF, (%)	63.5±5.5	63±7	0.3794	63.5±6.5	0.4065
Antiarrhythmic drug therapy, n (%)	2(6.67)	12(40.00)	0.0483	25(85.33)	<.0001

Patients with PAF received more antiarrhythmic medications (AAD) han the control group (*P* = 0.0483). There were no significant differences in age, gender, blood pressure, or left ventricular ejection fraction (LVEF) between the PAF group and the control group.

Patients with PersAF were significantly older (*P*<0.001) and were more likely had hypertension (*P* = 0.0137) compared with healthy control group. There was a significant difference in the use of antiarrhythmic drugs between the PersAF group and the control groups (*P*<0.001). There were no differences in the gender or LVEF between the PersAF group and the control group.

### Differential expression of four selected microRNAs

**Figure 3:**
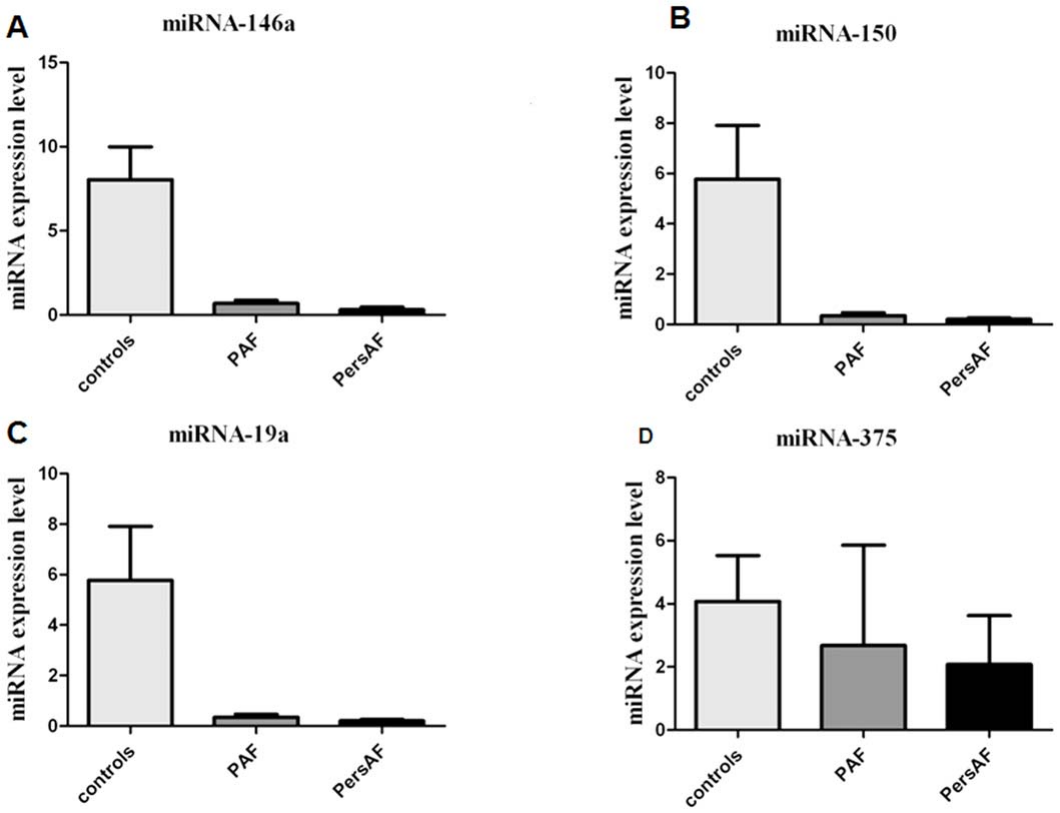
Absolute expression values of miR-146a, miR-150, miR-19a, and miR-375 in PAF patients (n = 30), PersAF patients (n = 30), and the control group (n = 30).

**Figure 4:**
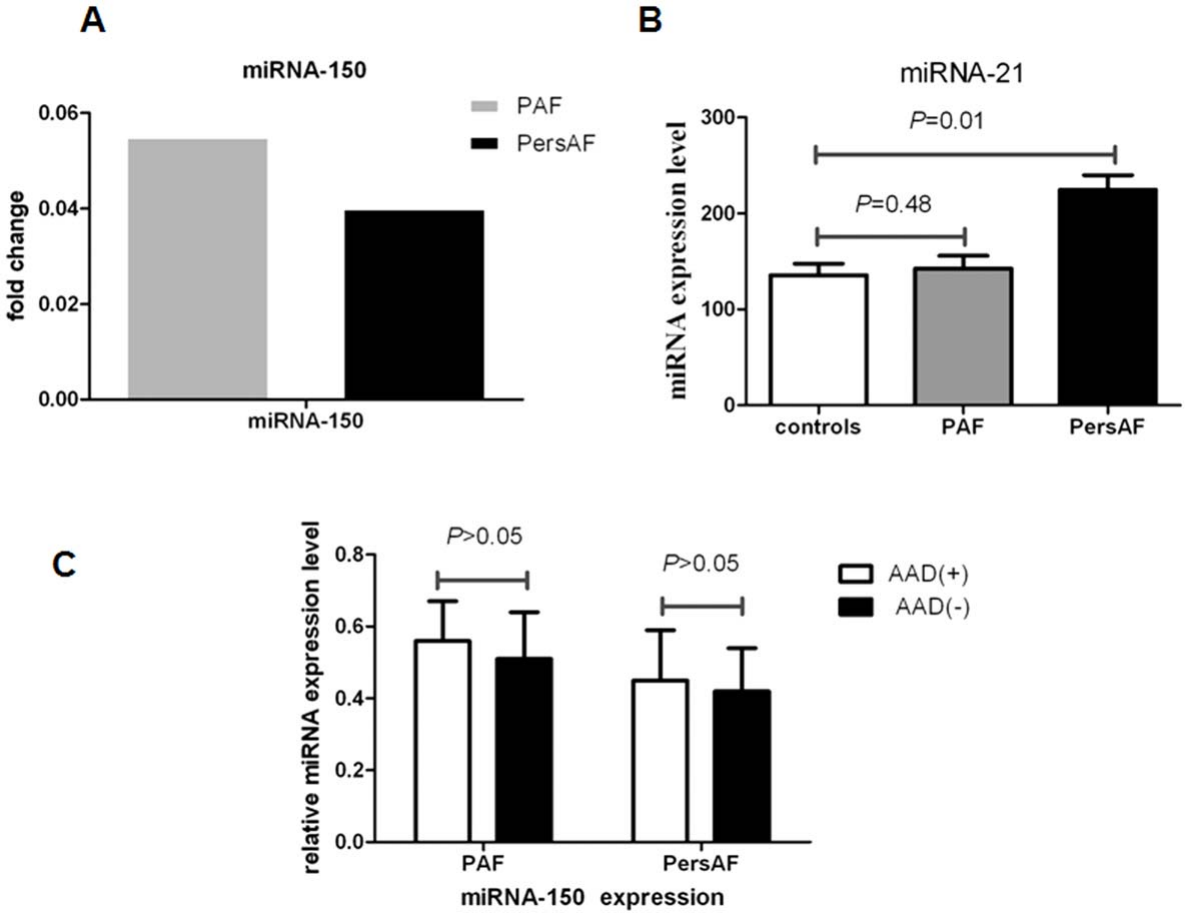
Expression levels of miRNA-150 and miRNA-21 in the AF patients. (**A**) Fold change of miR-150 relative to the control group in PAF and PersAF. (B) Plasma level of miRNA-21 in PAF patients (n = 30), PersAF patients (n = 30), and the control group (n = 30). (C) Expression levels of miRNA-150 in the AF patients with antiarrhythmic drug therapy (AAD+) and without (AAD−).

Four candidate microRNAs (miRNA-146a, miRNA-150, miRNA-19a and miRNA-375) were tested using an independent cohort of 90 plasma samples with qRT-PCR. The expression levels of these four microRNAs confirmed the existence of significant downregulation. Only the expression tendencies of miRNA-150, but miRNA-146a, miRNA-19a, and miRNA-375, correspond to the result of the in-depth analysis. (Fig. 3). MiR-150 demonstrated a pronounced change, with expression levels dropping by a factor of approximately 17 times in PAF patients relative to controls and a factor of approximately 20 in PersAF patients relative to controls (Fig. 4 A). We computed the median expression of miRNA-150 in each of the two groups using a logarithmic scale (*P*<.0001). The results suggested statistically significant differences between the levels of miRNA-150 among the healthy controls, PAF patients, and PersAF patients (Table 2). In addition, the presence or absence of antiarrhythmic drug therapy in AF patients had no relation to miRNA-150 levels (*P*>0.05). (Fig. 4 C)

**Table 2:** Significant differences of the levels of the 4 miRNAs.

	PAF vs Pers	PAF vs Control	Pers vs Control
	Sig	Sig	Sig
miRNA-146a	<0.0001	<0.0001	<0.0001
miRNA-150	<0.0001	<0.0001	<0.0001
miRNA-19a	0.258	<0.0001	<0.0001
miRNA-375	<0.0001	<0.0001	<0.0001

We detected the expression of miRNA-21 in the plasma samples of the three groups. We found no significant difference between the control group and the PAF group (*P* = 0.48). Expression was increased (∼0.8-fold) in the PersAF group (*P* = 0.01). (Fig. 4 B)

### Correlation between miRNA-150 expression and atrial fibrillation

There were significant correlations of the miRNA-150 expression in AF patients' LAD (r = −0.2, *P*<0.001) and age (r = −0.12, *P* = 0.006). To determine the overall correlation between miRNA-150 expression in patients and controls, we computed the levels of all miRNAs in both groups on a logarithmic scale. Age (*P*<0.001), LAD (*P*<0.001), and miRNA-150 concentration (*P*<0.001) were identified as independent predictors for the occurrence of AF. When data from the entire study group was entered into the logistic regression analysis, using AF as a predictor, reduced miRNA-150 expression levels (OR 1.96, 95% CI 1.5 to 3.57, *P*<0.001), age (OR 1.1, 95% CI 1.36 to 2.73, *P*<0.001), and LAD (OR 1.5, 95% CI 1.36 to 1.8, *P*<0.001) were independently associated with the presence of AF.

Because there was a significant difference in the expression level of miRNA-150 in the PAF and PersAF groups (*P*<0.001), we tested the use of miRNA-150 to discriminate PAF from AF. A group of 60 patients with AF (30 with PAF and 30 with PersAF) were included in the study. In the logistic regression model we did not observe significant associations between PAF and miRNA-150 (OR 1.02; 95% CI 0.82–1.13; *P* = 0.61).

### MiR-150 Expression Profile Is Correlated with Genes that involve in AF

As a next step, we tried to determine if miRNA-150 was linked to the pathogenesis of AF. One way to do this was to identify correlations in clinical samples between levels of miR-150 expression and that of important protein-coding genes involved in the pathogenesis of AF. We performed target prediction for miR-150 by using 4 Databases (TargetScan, miRanda, Starbase Clip-seq and miRDB) and identified the genes predicted by at least the three of the four databases. We found that at least 18 genes were related to AF. Among these, 11 genes were related to the inflammatory response system, 4 had a role in calcium ion channels, 2 were involved in apoptosis, and 1 related to fibrosis (Table 3).

**Table 3:** The Most Overrepresented Pathways for miR-150 targets According to KEGG*.

	KEGG Pathway Term	Count	*P* value	GENE NAMES
	hsa04150:mTOR signaling pathway	2	0.0218	EIF4E, EIF4B
Inflammation system	hsa04010: MAPK signalingpathway	4	0.0136	ARRB2, MAPK9, MAP2K4, PAK1
	hsa04910:Insulin signaling pathway	2	0.0347	PRKAR1A, EIF4E
	hsa04350:TGF-beta signaling pathway	1	0.0379	SP1
	hsa04012:ErbB signaling pathway	1	0.0391	CAMK2G
	hsa04310:Wnt signaling pathway	1	0.046	PRKCA
Apoptosis	hsa04210:Apoptosis	2	0.0215	IRAK2, PRKAR1A
		1	4.16E-03	CAST
calcium ion channels		1	1.24E-02	CAMK2G
		1	3.68E-02	KCNIP1
		1	0.0389	KCNIP1
fibrosis		1	0.0392	TPM3

* Count, number of potential target genes in the pathway. The gene name is presented as in NCBI at http://www.ncbi.nlm.nih.gov.

Inflammation is not only was a marker of incident AF, but is also mechanistically involved in the induction of atrial fibrillation [6], [7]. In our predicted target genes, IL-6 [32], [33], [34], IL-18 [35], TNF-α [36], and TGF-β [37] were reportedly increased in patients with AF significantly. Cytokines like these are produced by activated cells, usually monocytes and macrophages which also secrete miRNA-150 [38], in response to inflammatory stimuli. They are paramount in activating the inflammatory cascade and in the production of acute-phase proteins. One such acute-phase protein that is the center of much research is CRP [39], [40], [41]. These findings suggest a negative correlation between the expression of inflammatory cytokines and miR-150. We wondered if the magnitude of miRNA dysregulation correlated with the blood level of the inflammatory biomarker, CRP.

**Figure 5:**
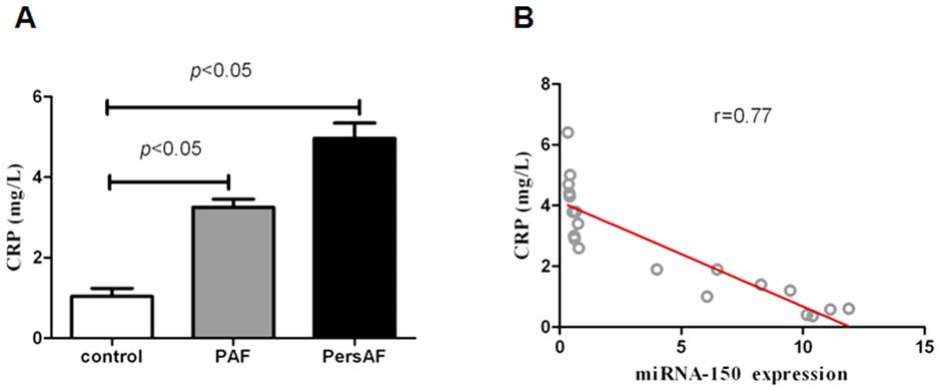
ELISA determination for plasma markers of sepsis and correlation with miR-150 expression in plasma are depicted in the graphs. (A) CRP measurements by ELISA in plasma. (B) A negative correlation was found between miR-150 and CRP.

We found that plasma CRP levels in patients with PersAF were higher than in patients with PAF, and levels in both AF groups were higher than the control group. MiRNA-150 had a high correlation with CRP. (Correlation coefficients up to 0.77 (Fig. 5).

## Discussion

Current methods for the diagnosis of AF are entirely limited to ECGs. However, the diagnostic performance of ECGs is unsatisfactory, particularly for the diagnosis of early-stage AF. Several features of AF, such as its brief duration, episodic frequency, and asymptomatic presentations, make its detection difficult with routine evaluation using pulse monitoring and ECGs [42]. Although 24 hour Holter ECG and 7 day Holter ECG will each increase the detection rate of AF, the long duration of monitoring involved makes this approach inconvenient to most early-stage asymptomatic AF patients [29]. Significant efforts have been made to identify a better plasma marker of AF, with limited success. Circulating micoRNA levels and aberrant expression of microRNAs in AF tissue has been identified [16]. Both be can used as biomarkers for diagnosis of acute myocardial infarction and heart failure. This has paved the way for further analysis of circulating microRNAs as biomarkers for predicting AF [14], [15], [43].

The present work demonstrated a specific set of miRNAs in AF patients that showed unique expression. The expression levels of miRNA-146a, miR-150, miRNA-375, and miRNA-19a were significantly downregulated in patients with AF. However, only miRNA-150 expression was characteristic enough to merit further evaluation. In our population of AF patients, we found significantly lower expression levels of miRNA-150 that were independently associated with age and LAD. We identified miR-150 as a predictor of AF (OR 1.96, 95% CI 1.5 to 3.57, *P*<0.001).

There were no differences between the control group and the theoretically earlier clinical stage PAF group. The expression of miRNA-21 in the plasma samples of the PersAF group was increased compared to controls. Recent evidence shows that miR-21 is an important regulator of fibrotic remodelling during AF [44]. It may play a major role in the later period of AF (PersAF) via the mechanisms of fibrosis. We were still unable to distinguish PAF from PersAF, possibly because of their similarities in classification and definition. The AF patients in this study included individuals without structural heart disease and some individuals with hypertension or CHD. Additional investigations with larger cohorts are needed to extensively evaluate.

Volker Rudolph and René P Andrié demonstrated that myeloperoxidase (MPO), an enzyme released by activated neutrophils and monocytes, is a crucial prerequisite for structural remodeling of the myocardium, and can contribute to the development of AF [7]. MiR-150 is abundantly expressed in monocytes [45], and most target genes in our study were related to the inflammatory system. This suggests that miR-150 may be involved in the induction of atrial fibrillation. Our study identified a high correlation between levels of miRNA-150 expression and CRP, a sensitive marker of systemic inflammation.

MiRNA-150 is not a heart-specific miRNA. Because our miRNA profiles are derived from plasma and not myocardial tissue, the miRNAs that become deregulated in AF might equally be derived from cellular populations outside the heart. Endothelial dysfunction and damage have been demonstrated in AF patients, and is a possible alternative source [46]. The increase in atrial fibrosis had been shown to correspond to an increase in conduction heterogeneity and AF vulnerability in animal models of CHF [47], [48], [49], [50] and in a transgenic mouse model of selective atrial fibrosis [49].

Overexpression of miRNA-150 can inhibit cellular proliferation and expression of smooth muscle α-actin (SMA) and collagen I [51]. MiRNA-150 also inhibits the activation of hepatic stellate cells (HSC) and the production of extracellular matrix (ECM), while regulating hepatic fibrosis [52]. We speculated that lower levels of miRNA-150 expression observed in AF patients may cause myocardial fibrosis and promote the formation of AF. Other miRNAs such as miRNA-21 [11], miRNA-133 and miRNA-590 [53], may also promote AF by stimulating cardiac fibrosis. Although our study confirmed that miR-133 is differentially expressed in PAF and PersAF patients, the expression level was very low (copy<10). The expression of miRNA-21 in the plasma samples was not significantly different. MiRNA-590 was not studied further at the qRT-PCR level, because it failed to meet our selection criteria.

A study has revealed a four-fold elevation of miR-328 in human atrial samples from AF patients [16]. However, in our study, the expression level of circulating miRNA-328 was not significantly different in AF patients compared to controls (*P* = 0.39). Y. Kuwabara et al [43] suggested that circulating cardiac miRNA-133 may originate from dead cells after myocardial infarction. The mechanism by which miRNAs are released in AF patients is not clear.

This study is limited by the small number of samples used in our analysis, which limits our ability to confirm the diagnostic power of microRNA signatures and their value for clinical testing in AF patients. Future prospective trials on larger patient cohorts are needed to establish the roles of these miRNAs in atrial fibrillation.

In summary, we firstly found that miRNA-150 level in plasma from AF patients was substantially lower than that from healthy people. Circulating reduced miRNA-150 was significantly associated with AF.
